# The P300 as a Marker of Waning Attention and Error Propensity

**DOI:** 10.1155/2007/93968

**Published:** 2008-02-12

**Authors:** Avijit Datta, Rhodri Cusack, Kari Hawkins, Joost Heutink, Chris Rorden, Ian H. Robertson, Tom Manly

**Affiliations:** ^1^Medical Research Council Cognition and Brain Sciences Unit, CB2 7EF Cambridge, UK; ^2^Hull York Medical School, York Hospitals NHS Foundation Trust, York YO31 8HE, UK; ^3^Arnold School of Public Health, University of South Carolina, Columbia, SC 29208, USA; ^4^Department of Psychology and Institute of Neuroscience, Trinity College, Dublin 2, Ireland

## Abstract

Action errors can occur when routine responses are triggered inappropriately by familiar cues. Here, EEG was recorded as volunteers performed a “go/no-go” task of long duration that occasionally and unexpectedly required them to withhold a frequent, routine response. EEG components locked to the onset of relevant go trials were sorted according to whether participants erroneously responded to immediately *subsequent* no-go trials or correctly withheld their responses. Errors were associated with a significant relative reduction in the amplitude of the preceding P300, that is, a judgement could be made bout whether a response-inhibition error was likely before it had actually occurred. Furthermore, fluctuations in P300 amplitude across the task formed a reliable associate of individual error propensity, supporting its use as a marker of sustained control over action.

## 1. INTRODUCTION

“Absent-minded” slips of action often result from the inappropriate production of an automatic or routine response [1]. Many of us will have repeatedly attempted to switch on light bulbs that we “know” need replacing, or automatically driven a familiar route when we were intending to go elsewhere. Although routine activities may be skilfully performed with little requirement for continuous control, there are occasions when such unsupervised actions can have serious consequences, from personal accidents to major disasters [2]. Moreover, the tendency to make such action errors significantly increases following traumatic brain injury, focal frontal lesions, and in some developmental disorders [3–12]. Here we examine whether time-locked EEG components may be sensitive to different states in which such errors are more or less likely to occur.

Slips of attention have been studied both in terms of predicting difficulties faced by clinical groups and in developing models of normal executive control over action. Norman and Shallice [13] and Shallice [10], for example, proposed an influential framework in which routine actions are controlled in a relatively automatic or stimulus-driven manner. Within this view, the expression of one behavioral sequence rather than another is governed by a competitive process determined by the strength of environmental triggers. Via such a system, apparently complex activities such as those involved in driving a car can be performed appropriately with little requirement for higher-level control. The second level of control, termed supervisory attention, is then proposed to modulate action selection if, for example, the most active behavioral sequence is inappropriate in relation to an overall goal. Such control is also experienced subjectively as effortful and conscious attention. More recently proposed frameworks draw similar distinctions. Dehaene and Naccache [14], for example, argue for a fronto-parietal circuit that acts as a “global workspace,” regulating more routine processes and which is associated with conscious effort. One set of conditions under which supervisory control is argued to be crucial is that presented in sustained attention tasks. In such tasks, the environmental triggers for goal-related behavior are reduced to a minimum, either by making the task “boring,” increasing the time over which a participant has to self-maintain a readiness to respond, and/or increasing the duration beyond a point of tedium [15–17]. The more successful a task is in reducing environmental support, the greater is its emphasis on the internal, or “endogenous,” maintenance of the appropriate processing stance.

Robertson et al. [9] developed a simple paradigm designed to assess self-maintained attention to current action. In the sustained attention to response task (SART), participants' watch-as-single digits are presented on a computer screen at a regular, invariant rate. They are asked to press a single button for each digit as it appears. The rhythmic nature of this response, coupled with the lack of selection, was designed to rapidly establish a relatively automatic, task-driven response. Periodically and unpredictably, however, a “no-go” target is presented to which no response should be made. In order to maximize the chances of not making an error, it has been argued, participants must try and counter the tendency to lapse into routine responding and maintain a high degree of control over action throughout the task. This brief and reliable task has proved to be sensitive to the frequency of everyday action lapses in traumatically brain injured patients [9] and in neurologically healthy volunteers [18].

The electroencephalogram (EEG) signal reflects brain activity including that which is in response to a specific environmental event. Such event-related responses are often difficult to separate from other activity on a trial-by-trial basis. If time-locked signals to many identical events are averaged, however, the unrelated signal tends to cancel out and the event related potentials (ERPs) emerge. The electrophysiological correlates of performance on tasks, such as the SART, that emphasise alternation between responding and not responding (termed “go/no-go” tasks) have been extensively examined [19–23]. The emphasis in such studies has been on differential responses to the presentation of the no-go stimulus relative to the go trial. Mäntysalo [23], for example, found increased amplitude of a negative component (N200) and a positive component (P300) on no-go trials a feature subsequently interpreted by Kok [22], and by Eimer [19] as reflecting response-inhibition processes. Jackson et al. [20] also found that the P300 component to the visual stimulus was more rapidly suppressed during no-go trials. These studies place emphasis on what happens after a “no-go” trial is presented. The focus here is on what happens *before* a no-go trial is unexpectedly presented. If, as has been argued, the ability to control action on no-go trials is determined by a *pre-existing attentive* state (sustained attention during the task), then it may be possible to assess this independently of overt behavior using ERP measures. Our hypothesis was that correct go trials that precede a correctly withheld response in a no-go trial should show evidence of this heightened attentive control relative to go trials that precede an error. A conceptual advantage of this approach lies in the degree to which other factors that might influence the ERP are controlled. In each case, the comparison is between correct go trials that are identical in terms of the stimulus presented (go), the response made (press), the instructional set (do not press for no-gos), and the probability of a subsequent trial being a no-go signal (1/8). If reliable differences emerge between trials that precede an action slip and those that do not, this can be interpreted with some confidence as being related to the attentional state of the participant under which subsequent errors are more or less likely.

There were cogent reasons for us to focus on the P300 ERP component as a likely predictor of errors in the SART go/no-go tasks. The P300 is a positive wave occurring approximately in 300 milliseconds following stimulus presentation [24, 25]. In contrast to some earlier components within the ERP, the P300 has been argued to reflect higher-level processes that are sensitive to task context, such as attentive selection [24, 26]. Increased P300 amplitude has been reported when participants detect that they have made an error in go/no-go tasks [27, 28], which may be interpreted in terms of error detection or the consequent establishment of a more attentional stance in which subsequent error probability is reduced. Further, studies have shown that the P300 is significantly reduced in survivors of traumatic brain injury, a group who have particular difficulty in avoiding errors on the SART [29–31].

In the current study, a group of neurologically healthy volunteers performed multiple blocks of the SART task to establish whether variations in P300 amplitude were associated with action errors in the SART. For each participant, the 250 *no-go* trials from the 10 blocks of the SART were first indexed and sorted according to whether the participant had made a commission error, by incorrectly pressing the response key, or had correctly withheld the response. For each of these categories, the visual ERPs to go trials that immediately *preceded* these no-go trials were then averaged first for each participant and then for the group of 25 participants as a whole. From previous studies, we anticipated sufficiently high error rates in this group to allow a reasonable comparison between events prior to a correct no-go trial and prior to an action error.

Previous studies have shown that SART is relatively reliable in picking up enduring individual differences in error propensity. In addition to the hypothesis that relatively high or low P300 amplitude would be associated at a *within-subject* level with different subsequent error rates, we therefore further hypothesized that individual differences in the degree to which the P300 component was maintained across all of the go trials would be associated with individual differences in error rates.

For both analyses, there were advantages if gross individual differences in P300 amplitude (e.g., due to the quality of electrode contact, skull thickness, etc.) could be reduced. To this end, we expressed P300 in proportion to that of an earlier ERP component, the P200 (P200 : P300 ratio). The P200 should be subject to the same intersubject differences affecting absolute amplitude but, in being thought to reflect more perceptual aspects of the neural response, less likely to be modulated by current attentional engagement with the task. For this reasoning to be valid, it would be necessary to additionally demonstrate *in the current task* that variations in the P300 are related to subsequent error while variations in the P200 are not.

The hypotheses can therefore be summarized as follows.

1. Having first grouped no-go trials according to whether or not an error occurred, the average amplitude of the P300 on prec*eding go trials* will vary in relation to the outcome on those subsequent no-go trials. The earlier and more perceptual P200 will not.
2. If so, this will allow us to reduce gross between-subject differences by expressing P300 amplitude relative to that of the P200 (P200 : P300). Averaged across the group, we then predict that the “normalized” P300 value will differ between go-trials preceding an error and those preceding a correct no-go trial.
3. In addition to those go trials that immediately precede no-go trials, we would expect the degree to which the normalized P300 amplitude is maintained across the task as a whole to reflect error rates. Specifically, in a correlational analysis, the mean normalized P300 amplitude (P200 : P300) across *all* go trials will be associated with individual differences in error propensity.

## 2. MATERIALS AND METHODS

### 2.1. Participants

Following ethical committee approval, 25 neurologically healthy right-handed volunteers (13 women and 12 men, age range 20–47) gave informed consent for their participation in the study.

### 2.2. Electrophysiological recording and averaging

EEG recordings were made from 3 midline sites (Fz, Cz, Pz) using silver/silver chloride electrodes (Grass). Four additional electrodes were applied for eye blink and movement monitoring, grounding, and reference. The electrodes were referenced to the right mastoid during recording. The horizontal electro-oculogram electrodes were referenced to each other. The EEG and EOG signals were amplified with a bandwidth of 0.05–100 Hz. The digitization rate for the analogue-to-digital conversion was 500 samples per second. Prior to averaging, artefact rejection was performed on the data to discard epochs in which amplifier saturation, eye movements, blinks or excessive muscle, or movement artefacts occurred. The same rejection criteria were used for all participants. In some cases, the rejection values for eye artefacts were individually adjusted, to correct for individual differences in amplitudes of artefacts and EEG. This procedure resulted in an average rejection of no more than 2% of the trials for each of the 25 subjects included in the analysis. Electromyogram signals (EMG) in the responding hand were monitored using bipolar silver/silver chloride electrodes from an index finger flexor (first dorsal interosseous muscle) and an index finger extensor (extensor indicis). EEG was amplified 20 000 fold, EMG 1000 fold, and EOG 2000 fold using AC coupled amplifiers (Biopac Systems Inc., Santa Barbara). Filtering was 10 Hz–5 KHz, 1–35 Hz, and 0.05 Hz–100 Hz for EMG, EEG, and EOG, respectively. Full-wave rectification of the EMG was performed digitally. All data was digitized at 500 Hz, indexed for go, no-go stimulus, correct and incorrect response, archived, and averaged offline using a purpose-written averaging program. For stimulus-locked averages, the P300 was defined as the maximum positive peak amplitude between 250–450 milliseconds after stimulus presentation. Latencies of peaks were clearly identifiable in each case.

### 2.3. Behavioral task: the sustained attention to response test

The task [32] was presented on a Dell Latitude laptop computer isolated from the mains supply. On each trial, a single digit (1–9) was selected at random and presented for 250 milliseconds, followed by a mask for 900 milliseconds, at the center of the 185 mm × 245 mm screen. Participants, who were at a comfortable viewing distance from the screen (around 40 cm), were asked to press a mouse button with the index finger of their preferred hand as quickly as possible after each digit presented, with the exception of 3, to which no response should be made. They were asked to press the button “as quickly but as accurately as possible” following the onset of the trial. The randomization meant that 25 no-go trials (3 seconds) appeared unpredictably amid 200 go trials (all digits other than 3) in each block. Each participant completed 10 blocks with the opportunity to rest from the task between each.

**Figure 1:**
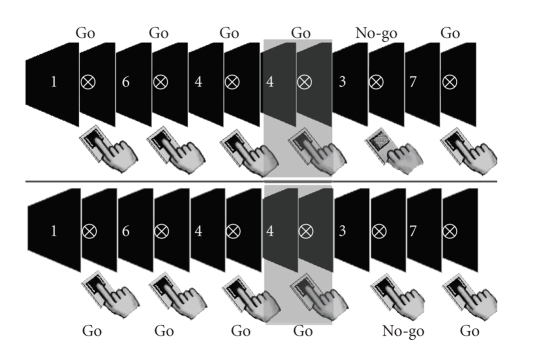
Selection of trials for the main comparison. Each figure represents the sequence of events in the SART where go trials are defined by any digit between 1 and 9 (except 3) and the no-go target by the 3. In each sequence, the participant is responding correctly to go trials. In the upper panel, the presentation of the target is followed by a correctly withheld response. In the lower panel, by an error, the correct go trials *prior to* these no-go signals (highlighted) form the basis of the comparison.

Testing took place in a quiet, darkened room that was free from distraction. The total testing session, including setting up and removing the recording electrodes, lasted for approximately 3 hours. Figure 1 illustrates the sequence of events in the task and the two types of go trial (defined by immediately subsequent no-go trial error) that inform the main ERP comparison of this study.

## 3. RESULTS

### 3.1. Performance on the task

The participants completed 10 blocks of the SART, comprising 2000 go trials and 250 (11.1%) randomly intermixed no-go targets. The participants correctly withheld their responses to 147.72 (59%) of the 250 no-go trials (SD 16.12) and made errors of commission on an average of 102.28 (41%) of these trials (SD 16.105). As is common, errors of *omission* (i.e., not pressing the response key on go trials) were very rare, occurring on an average of 0.55 of the 2000 go trials (0.061%, SD 0.17%).

### 3.2. ERPs to the visual stimulus prior to a correct no-go trial and prior to an action error

Previous behavioral studies with the SART suggest that, other than in severely brain injured individuals, correct responses on no-go trials are likely to outweigh errors of commission. As reported above, this was the case with the healthy participants tested here. Correct responses accounted for about 60% of the no-go trials with around 40% attracting action errors. This error rate is somewhat highly compared with previous studies and may be related to the presentation of 10 consecutive blocks, rather than the more conventional single block. This higher rate is, however, to our advantage in comparing pre-error and pre-correct go trials. With both categories yielding between 80 and 170 trials per person (pre-error mean = 102.28, SD 16.11, range 80–147, pre-correct mean = 147.72, SD 16.10, range 103–170), there are sufficient numbers for noise to tend towards zero in the averaged signals for each participant in both categories, with these values then being again averaged across the group. Any differences between the waveforms should *not* therefore be attributable to a disparity between the overall number of error and correct trials. However, the risk of this unlikely confound is further reduced by our focus on a single wave, the P300, as the component that should show a difference. If it is the P300 which is indeed different while other components are broadly equivalent, it is less likely that *differential* amounts of noise, which would be distributed across the signal, would have such a specific effect.

**Figure 2:**
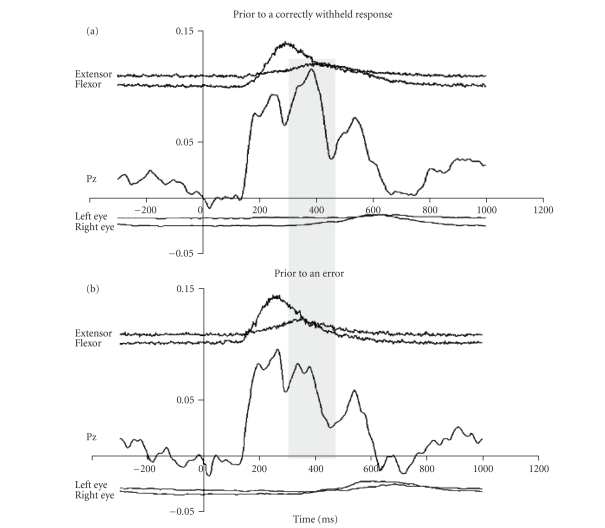
The difference between go trials preceding a correct or erroneous no-go trial. Each figure shows EEG (at Pz), finger muscle activity (extensor/flexor), and eye movements (left and right) averaged across all available relevant trials. The crucial difference between these go trials appears to be in the amplitude of the P300 ERP peak, highlighted in the grey band.

The Pz ERPs for go trials before an error and before a correct response suppression are presented in Figure 2 below. Figure 2(a) shows the pattern that preceded a correct no-go trial while Figure 2(b) shows the pattern preceding an action error. Each panel shows (from top to bottom) averaged rectified agonist and antagonist muscle electromyogram (EMG), averaged scalp electroencephalogram (EEG; at Pz), and averaged electrooculogram (EOG; used in controlling for eye movements).

In both Figure2(a) and Figure2(b), a triphasic response is observed in Pz EEG with peaks of each wave occurring at similar latencies (270 milliseconds, 384 milliseconds, and 544 milliseconds in Figure 2(a), and 250 milliseconds, 388 milliseconds, and 542 milliseconds in Figure 2(b)). The amplitude and latency of the positivity between 200–300 milliseconds after the onset of the trial (P200) is strikingly similar to the two trial types. Given that these go trials are effectively identical, other than in what *subsequently* happens, it is perhaps not surprising that the early perceptual components of the ERPs are so similar. As discussed above, we therefore exploited this stable feature in order to allow a comparison of the P300 components that was relatively free from the influence of noise and inter-subject variables such as signal intensity. The amplitude of the P300 component was therefore expressed as a ratio of the P200 amplitude for each participant averaged across the two trial “types” illustrated in Figure 1.

**Figure 3:**
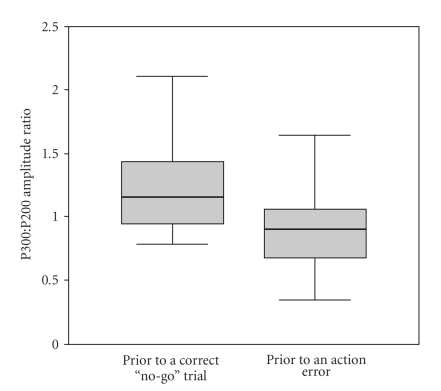
The difference between go trials preceding a correct or erroneous no-go trial. The figure shows a boxplot for the P300 : P200 amplitude ratio for the two “types” of go trial. Each shows the median (heavy line), interquartile range (shaded area), and total range for the 25 volunteers. Prior to a correct no-go trial, the median normalized P300 amplitude exceeds even the interquartile range of that seen in go-trials prior to an no-go error.

Comparison of the P300/P200 ratio between trials preceding an action error (mean 0.92, SD 0.33, *n* = 25) and trials preceding correct withholding of the response (mean 1.28, SD 0.48) reveals a robust and statistically significant difference (t(24) = 3.63, *P* < .001). Moreover, Figure 3 shows that the median P300/P200 ratio prior to an action error falls below even the interquartile range of the P300/P200 prior to a correct no-go trial.

In both Figures 2(a) and 2(b), EOG traces are flat until 600 milliseconds after presentation of the visual stimulus. This lack of contamination of eye movement allows confident interpretation of EEG traces and P300/P200 ratios that we have obtained. Although similar responses were seen at Cz and Fz sites, these were less compelling in magnitude and did not reach statistical significance for this group size. For brevity we will therefore focus on the Pz results in subsequent analyses (see later for discussion).

In summary, in two groups of go trials which are identical other than in what occurs on the *subsequent* no-go trial, there appears to be a difference reflected in the P300 at Pz which is related to the probability of a subsequent error. When this amplitude is relatively low, errors are more likely. For reasons outlined in the introduction, therefore, it is tempting to argue that this component is reflecting some form of enhanced attention to the stimulus/task which makes errors less likely.

### 3.3. Individual differences in error propensity

In the previous section we considered only those go-trials that immediately preceded no-go trials. If it is the case, as the results suggest, that an increased P300 amplitude is associated with more attention and fewer errors, it might be expected that the mean value of this marker across the whole task could reflect an individual’s capacity to maintain an attentive state and overall “resistance” to inhibition errors. To examine this, we examined the Pearson correlation between each participant's (averaged) normalized P300 amplitude across all of the 2000 go trials in the task and their overall commission error rates on no-go trials.

**Figure 4:**
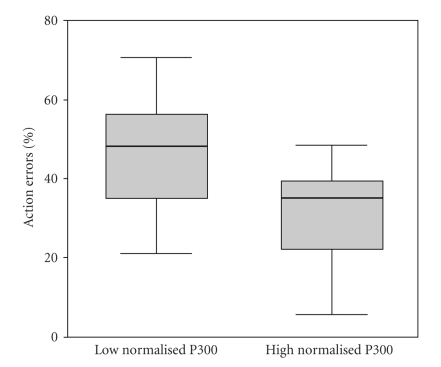
Propensity to error is associated with the maintenance of the P300 : P200 ratio across the task. The boxplot shows error frequencies for participants with high or low mean P300 values (defined by a median split of the total participant group), respectively.

The relationship was statistically significant (Pearson's *r* = −0.46, *P* < .05), the lower the relative average amplitude of the P300, the more action lapses a particular participant was prone to make. This relationship is further illustrated by the division of the participants into “high” and “low” relative P300 groups based on a median split. As can be seen in Figure 4, 12 participants with low Pz P300/P200 ratio values (between 0.34 and 1.07) had a mean error rate of 47% (SD 15.4%) while 13 participants with high Pz P300/P200 ratio values (between 1.09 and 2.25) made significantly fewer errors (32.5% (SD 13.4%); t(23) = 2.51, *P* = .02).

### 3.4. Error detection and reaction time effects

So far we have seen that a reduced relative amplitude of the Pz P300 is associated with a higher probability of an error on a subsequent no-go trial and, over all of the go trials in the task, associated with increased no-go error propensity. We have so far interpreted this in terms of reflecting waning attention to the stimulus and task. However, before we can do that with confidence, there are a couple of potential confounds that should be addressed. These are “contamination” of our go trial ERPs with processes related to the detection of a *previous* error and the possibility that trials preceding errors had rather different reaction times to those preceding correct no-go trials.

The SART is a continuous task. If one has a high *overall* rate of errors on no-go trials, it is more probable that any given go trial might have occurred *after* a previous error, as well as possibly *preceding* other errors. If one made errors on 100% of no-go trials, for example, all but the first go trials may be considered to have “followed” an error. This is important because increases in the P300 have been associated with error detection, albeit that this is a feature that appears to be relatively short lived in the ERP trace [27, 28, 33]. It is possible, therefore, that the relationships so far reported between Pz P300 amplitude and subsequent error and overall error rates are mediated by error detection factors, although it should be noted that, were this the case, the direction of this relationship would be reversed (more errors = higher P300 amplitude). To examine this possibility, we compared the mean Pz P300/P200 ratio for those go trials that immediately preceded *no-go trials,* with the average for all go trials. Go trials that occur immediately before a no-go trial tend, by definition, to be as distant from a previous no-go trial as it is possible to be within the task and are therefore *less* likely to be influenced by error detection processes triggered by a previous mishap. If our previously reported correlation was substantially due to error-detection processes, we would expect the relationship between the P300 amplitude in *these* trials and overall error rates to be reduced. In fact, if anything, it was enhanced (Pearson's *r* = 0.57, *P* < .01).

Finally, we investigated whether the predictive qualities of the normalized Pz P300 may be mediated by reaction time (RT) differences. The relationship between mean RT to go stimuli and error rates across subjects did not, however, reach statistical significance (*r* = −0.281, *P* = .174), meaning that, in this group, we could not predict errors on the basis of how fast individuals were responding. In addition, there was no relationship between mean RT of participants and mean amplitude of their normalized P300 response (*r* = 0.1, *P* = .455, *n* = 25), further suggesting that the relationship between individual error propensity and P300 amplitude was not mediated by response speed differences.

## 4. DISCUSSION

In this study, we asked participants to perform a simple go/no-go tasks in which no-go targets appeared infrequently and unpredictably within a random sequence. Previous research has suggested that this task is sensitive to everyday absentminded lapses in people with brain-injuries and healthy participants. Furthermore, there is evidence to suggest that success on no-go trials is related to how well people are able to maintain active attentive control over their responses, rather than allowing them to be “driven along” by the repetitive, regular pacing. The basis for this study was that, if there is a fluctuating state of attention allocation in which errors are sometimes more probable and sometimes less, we might be able to see this within fluctuating electrophysiological signal before the critical no-go target has even appeared.

The results were consistent with this view. From the random sequence of trials in the task, we first found go trials that happened to have occurred before a no-go trial. We then divided these into those that had been followed by a correct response suppression and those that had been followed by an error. The EEG was then averaged for each grouping, time-locked to trial onset. It is again important to stress that, from the participants' perspective, trials preceding no-go signals hold no special status, indeed, any given go trial is around 8 times more likely to be followed by another go trial than a no-go trial. The ERPs on go trials had a characteristic triphasic pattern. Given that the trials are perceptually indistinguishable, it was not surprising that the early perceptual response in the EEG was similar whether the go trial occurred before an error or a correct no-go trial. A substantial difference was, however, apparent in the P300. When its amplitude was relatively low, it was associated with increased errors on subsequent no-go trials. When its amplitude was relatively high, participants were more likely to succeed in withholding their responses on subsequent no-go trials. As might be expected from this finding, the degree to which the amplitude of the P300 was maintained across the task was associated with individual error propensity among the participants.

It seems, therefore, that the P300 formed an electrophysiological marker of *something* that is probabilistically associated with subsequent error. It is tempting to view this as a fluctuating “top-down” goal-directed signal which, if it could speak, would be saying things like “watch out, don't press on the no-go trial, don't get distracted, keep focusing on the task, and so forth.” However, the averaging of the ERPs to the onset of each trial makes it less likely that we are directly sampling the intensity of such a signal. Instead, we are more probably detecting the *consequence* of that maintained stance in the attention/decision making allocation to each digit. In the SART, the really important presented digit is the one nominated as the no-go target. The others just mean that the current trial is not a no-go target and, when no-go trials are rare, arguably this encourages a stance in which evaluation becomes rather scant (and in which commission errors are more likely). This would be reflected in the reduced P300 to each digit presentation. The influence of a maintained goal-directed stance would be to resist this and encourage more active trial-by-trial decision making about the response with reference to the digit. This would be reflected in increased digit-onset locked P300 amplitude. The results are therefore consistent with many previous studies associating the P300 with increased attention to a particular stimulus (e.g., [19–23, 32]). The novel feature or argument here is in the relation of this individual stimulus processing to some more generally maintained executive stance to the task. More simply, it might be expressed as If there is a good attention at trial n (high P300), then it is more likely that there will be good attention at trial *n* + 1—which will be particularly useful if it happens to be a no-go trial.

There are a number of confounds or different interpretations of these findings which we have attempted to address. The first is that the results are an artefact of the different number of pre-error and pre-correct trials delivered to us by the participants. The actual number of trials contributing to the averages was, however, relatively high (between 80 and 147 for pre-error trials and between 103 and 170 for pre-correct trials). The averaging process should, therefore, have had a reasonable opportunity to reduce the contribution of random noise to near zero levels, and therefore a *differential* contribution of noise to the comparison should be minimal. In addition, and assuming that noise would be temporally as well as randomly distributed, the inference is strengthened by our focus on the P300 and the lack of marked difference in other components within the ERPs. Finally, in this respect, we further minimized the risk by expressing the magnitude of the P300 as a ratio of the P200. This process should further cancel any noise difference (in that the P200 should be equally susceptible) as well as offering other advantages in terms of normalizing the response. A second concern was that the P300 association with error was mediated by *previous* error detection. The observations that error detection has generally been associated with an *increase* in P300 (rather than the *decrease* that we see here associated with more errors), and that the “error-signal” is a rather short-lived phenomenon [34–36] both suggest that this account is unlikely. Furthermore, by comparing the correlation with overall error rates in go trials that immediately preceded no-go trials (which are as “far as you can get” from a previous no-go trial and hence error) with go trials in general, we found the P300 magnitude was increased, not decreased, with remoteness from an error. Finally, we found no significant differences in reaction times that could account for the results.

Despite this, we still need to be cautious. The statistically significant differences and correlations that we report are all from the Pz region. Although they were broadly in a consistent direction, the differences at Cz and Fz were less impressive. However, the site of biggest ERP signal difference may not be obviously connected to the origins of that difference and it seems improbable that the prefrontal cortex is not in some way involved in the allocation and maintenance of attention [32, 37–40]. It is also true that a plethora of functional imaging and other results now suggest that parietal regions tend to be coactivated with those of the dorso- and ventro-lateral prefrontal cortex in tasks requiring effortful or conscious processing [14]. While the current study may have little to say about the location(s) of the sources of the observed ERP differences, other studies may be more useful guides. Robertson et al. [32] examined ERP correlates of go no-go task performance in head-injured and healthy participants. As with our study, they found no significant differences between no-go and visually identical go trials in the early perceptual components in the ERP (up to and including the 200 milliseconds bin) in either group. For the healthy participants, increased amplitude at P300 did differentiate the trial types and was interpreted by the authors as reflecting increased attention and/or the launching of an inhibitory signal to prevent a response. In this respect, the healthy participants showed a greater differential response to no-go trials than the patient group, which may be reflected in their relatively lower error rates. Interestingly, in terms of our current discussion, Roche et al. identified two components in their P300. The P300 was reported to be maximal at the frontal electrode site whilst the slightly later P300b was of greater magnitude and most apparent at the Pz site. It is possible to question whether the common coactivation of frontal and parietal regions in effortful tasks which is commonly seen in functional magnetic resonance imaging (fMRI) studies reflects the simultaneous engagement of a large distributed network or whether, for example, parietal activity may be a secondary consequence of frontal activation. The combination of the good temporal resolution of ERPs and the spatial resolution of fMRI may be necessary to further address this question.

Electrophysiological measures such as this provide one route out of the conceptual circularity inherent in some purely behavioral analyses. Errors on the SART have previously been attributed to the poor maintenance of attention with that poor maintenance being marked by the occurrence of the error. This is a reasonable but circular argument that requires additional measures such as the frequency of attention problems in everyday life, self-reports of “task unrelated thought” propensity, and the effect of cues to maintain attention, if it is to be sustained [41]. An alternative, though not mutually exclusive, account might emphasise response inhibition efficiency as contributing towards errors in the task. Following Logan et al. [42] we might therefore view success or failure on a no-go trial as depending upon the outcome of a race between the erroneous “go” response and an inhibitory signal launched at the start of the trial. We would know if the internal “stop!” signal was a good or poor competitor based on the number of errors made and we would explain the number of errors made based on the hypothetical speed of this signal. Again, independent measures of response inhibition from other tasks or from everyday life would be required to avoid circularity. The advantage of the electrophysiological approach used here is that we can see that there is some influence at work *before* the critical no-go trial has been presented. Whether or not a race model is accurate or appropriate (and there are good reasons to believe it is both), the results suggest that there is *something* in place biasing the odds of that race before it has begun. This seems to chime with everyday experience of inhibitory failures. Returning to the light bulb example, if one enters the dark room thinking “Concentrate… habit tells you to switch on the light but you know that, in this case, it will not help!”, then—with luck—the action error is less likely.

This issue is not trivial as there are, as discussed, many clinical groups said to suffer from inhibitory deficits. In addition to the possibility of tweaking the efficiency of inhibitory control, perhaps pharmacologically, the results suggest that other interventions could serve to reduce the consequences of inhibitory difficulty. These would include programs designed to help people recognize situations in which a more attentive stance might offset inhibitory slips, training in maintaining such a stance, and the use of cues to externally support such maintenance when necessary. These programs would have application in rehabilitation of neurological patients and also assist situations where prolonged vigilance is vital such as in industrial or military scenarios. Although tasks such as the SART may be somewhat artificial models of aspects of everyday situations, their value lies in allowing close, controlled analysis of cognitive failures and, therefore, in refining understanding and evaluating interventions. They are also, in their repetitive structured way, compatible with the averaging over multiple similar events necessary for ERP analysis. We conclude that identifying EEG markers, such as the P300, which appear to reflect a well maintained top-down stance to a task therefore has multiple potential benefits in predicting and preventing potentially catastrophic errors in civilian and military life.
